# Opposing Roles of GSK3α and GSK3β Phosphorylation in Platelet Function and Thrombosis

**DOI:** 10.3390/ijms221910656

**Published:** 2021-09-30

**Authors:** Samantha F. Moore, Ejaife O. Agbani, Andreas Wersäll, Alastair W. Poole, Chris M. Williams, Xiaojuan Zhao, Yong Li, James L. Hutchinson, Roger W. Hunter, Ingeborg Hers

**Affiliations:** 1 School of Physiology, Pharmacology and Neuroscience, University of Bristol, Biomedical Sciences Building, University Walk, Bristol BS8 1TD, UK; Samantha.f.bull@gmail.com (S.F.M.); ejaife.agbani@ucalgary.ca (E.O.A.); j.a.wersall@gmail.com (A.W.); a.poole@bris.ac.uk (A.W.P.); chris.m.williams@bris.ac.uk (C.M.W.); jenna.zhao@bris.ac.uk (X.Z.); yong.li@bris.ac.uk (Y.L.); lawrence.hutchinson@bris.ac.uk (J.L.H.); roger.hunter@nhsbt.nhs.uk (R.W.H.); 2 Department of Physiology and Pharmacology, Cumming School of Medicine, University of Calgary, Calgary, AB T2N 1N4, Canada; 3 NHS Blood and Transplant, North Bristol Park, Filton, Bristol BS34 7QH, UK

**Keywords:** GSK3, PI3 kinase, Akt, integrin activation, P-selectin expression, thrombosis, knock-in mouse models, p110β, phosphorylation

## Abstract

One of the mechanisms by which PI3 kinase can regulate platelet function is through phosphorylation of downstream substrates, including glycogen synthase kinase-3 (GSK3)α and GSK3β. Platelet activation results in the phosphorylation of an N-terminal serine residue in GSK3α (Ser21) and GSK3β (Ser9), which competitively inhibits substrate phosphorylation. However, the role of phosphorylation of these paralogs is still largely unknown. Here, we employed GSK3α/β phosphorylation-resistant mouse models to explore the role of this inhibitory phosphorylation in regulating platelet activation. Expression of phosphorylation-resistant GSK3α/β reduced thrombin-mediated platelet aggregation, integrin α_IIb_β_3_ activation, and α-granule secretion, whereas platelet responses to the GPVI agonist collagen-related peptide (CRP-XL) were significantly enhanced. GSK3 single knock-in lines revealed that this divergence is due to differential roles of GSK3α and GSK3β phosphorylation in regulating platelet function. Expression of phosphorylation-resistant GSK3α resulted in enhanced GPVI-mediated platelet activation, whereas expression of phosphorylation-resistant GSK3β resulted in a reduction in PAR-mediated platelet activation and impaired in vitro thrombus formation under flow. Interestingly, the latter was normalised in double GSK3α/β KI mice, indicating that GSK3α KI can compensate for the impairment in thrombosis caused by GSK3β KI. In conclusion, our data indicate that GSK3α and GSK3β have differential roles in regulating platelet function.

## 1. Introduction

Phosphoinositide 3-kinase (PI3K) activation occurs downstream of multiple receptors involved in regulating platelet activation, ultimately controlling both thrombosis and haemostasis [1,2,3]. This has led to an interest in not only examining how this pathway can be targeted for anti-thrombotic therapies but how platelet function may be altered in the clinic in patients using drugs directed against this pathway for the treatment of cancer and neurological diseases.

PI3 kinase mediates its effect by the generation of phosphatidylinositol 3,4-bisphosphate (PI3,4P_2_) and phosphatidylinositol 3,4,5-trisphosphate (PI3,4,5P_3_), resulting in protein recruitment and activation of downstream signalling pathways. Proteins recruited in human platelets range from adaptor molecules (e.g., Dual Adapter of Phosphotyrosine and 3-Phosphoinositide 1, DAPP1) and small G-proteins (e.g., Ras GTPase-activating protein 3, Rasa3) to kinases (e.g., Ak strain transforming/protein kinase B, Akt/PKB) [4,5], and many of these contribute to PI3kinase mediated platelet function [6,7,8,9,10,11,12]. Activation of Akt kinase leads to phosphorylation and inhibition of Glycogen Synthase Kinase α/β (GSK3α/β) kinase activity.

The *GSK3* gene family encodes two highly conserved kinases, 47kD GSK3β and 51kD GSK3α. Both are ubiquitously expressed, multifunctional serine/threonine kinases [13] with both common and non-overlapping cellular functions, making them key regulators of numerous signalling pathways [14]. As such, they are believed to be important regulatory enzymes in many diseases and disorders such as cancer and aging, immune disorders, metabolic disorders (atherosclerosis, diabetes, and heart disease), neurological disorders, and thrombus stability [15]. GSK3α/β are unusual in that phosphorylation of an N-terminal serine results in a decrease in kinase activity towards substrates [16,17]. This occurs due to GSK3α/βs’ preference for target proteins that are pre-phosphorylated at a “priming” residue. This primed residue allows substrates to bind to a docking site on GSK3α/β that is adjacent to the catalytic cleft, amplifying the efficiency of substrate phosphorylation. If GSK3α/βs are phosphorylated at the N-terminal serine by effectors, this leads to the formation of a primed pseudo-substrate that binds to the docking site, blocking “primed” substrate binding [18]. GSK3α/β are generally active in a resting cell (constitutively active) and are therefore commonly “inactivated” by phosphorylation in response to cell stimulation. Their requirement for priming phosphorylation means that substrate phosphorylation is also regulated by the activity of the priming kinase.

Previous work examining the role of GSK3α/β in regulating platelet function has predominantly relied on the use of a single-allele knock-out or pharmacological inhibition [8,9,19,20]. Initial studies by Li et al. [19] demonstrated enhanced platelet responses to the thrombin receptor Protease Activated Receptor 4 (PAR4) peptide AYPGKF in platelets from single-allele GSK3β KO mice and reduced survival time upon collagen/epinephrine injection, suggesting GSK3β activity negatively regulates platelet function. This hypothesis was in agreement with human platelet hyperresponsiveness to PAR1 peptides in the presence of a GSK3 inhibitor [9,19,21]. Further support for a role of GSK3 in modulating platelet function was implied by the ability of GSK3 inhibitors to rescue the effect of p110β PI3kinase/Akt deficiency on platelet function [8,22,23,24]; Laurent et al. [22] demonstrated an important role of p110β PI3kinase in thrombus stability at a high shear rate, which was restored by pharmacological inhibition of GSK3. Furthermore, impaired thrombin-mediated platelet aggregation and platelet spreading due to Akt3 deficiency was rescued by GSK3 inhibitors [8,23]. The contribution of GSK3 to collagen-mediated platelet function is less clear, as different effects of GSK3 inhibitors on GPVI-mediated platelet function have been reported [8,9,20]. Both single-allele knock-out and pharmacological inhibition have demonstrated interesting roles for GSK3α/β in platelet activation but address the role of total GSK3α/β activity rather than the reduction in GSK3α/β activity towards substrates that results from GSK3α/β phosphorylation upon physiological platelet stimulation. In platelets, GSK3α/β phosphorylation is mediated by both a PI3K-independent pathway involving Protein Kinase C α (PKCα) and the PI3Kβ/Akt pathway [9].

In this study, we aimed to identify the role of phosphorylation of the individual paralogs GSK3α and GSKβ. To achieve this, we employed mouse models in which the inhibitory N-terminal serine of GSK3α and/or GSK3β was changed to encode a non-phosphorylatable alanine residue. This revealed that GSK3α and GSK3β phosphorylation have divergent roles in regulating platelet function.

## 2. Results

### 2.1. GSK3α and GSK3β Expression in Human and Mouse Platelets

**Figure 1:**
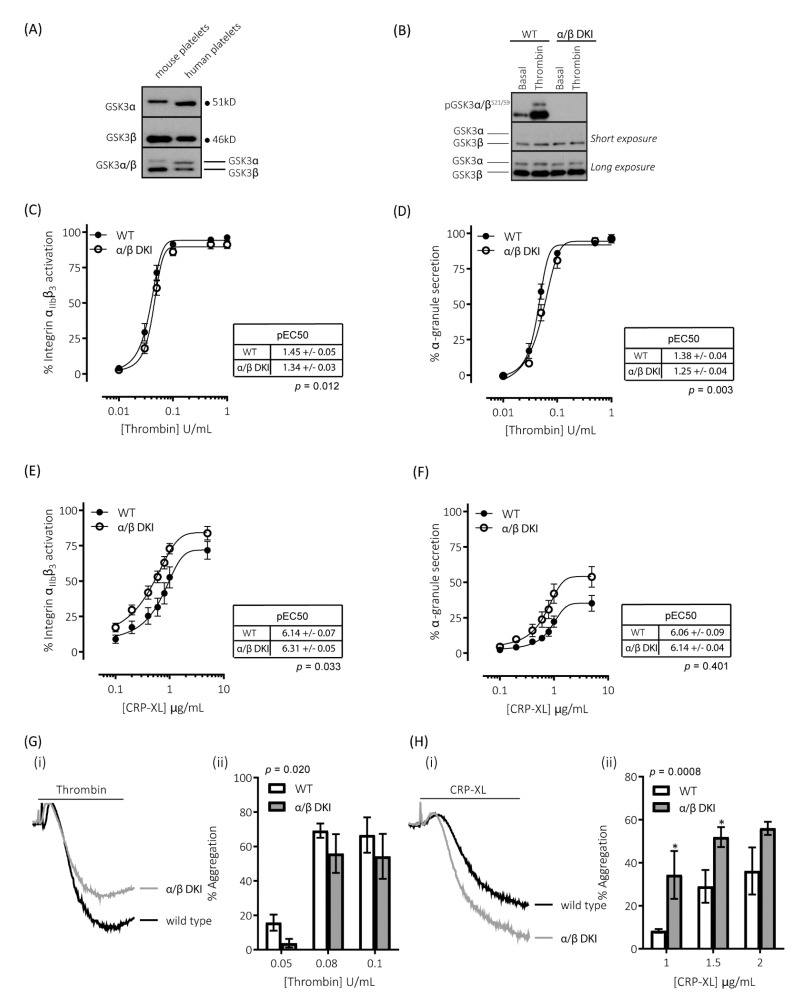
**Platelets expressing phosphorylation-resistant GSK3 have differentially altered responses to thrombin and CRP-XL**: (**A**) Immunoblotting of human and mouse platelet lysates demonstrating the expression of both paralogs of GSK3. GSK3α was observed to run at ~51kD and GSK3β at ~46kD. (**B**) Platelets from mice expressing phosphorylation-resistant GSK3 (α/β DKI) were found to express both GSK3α and GSK3β at levels equivalent to platelets from wild-type controls (WT). However, phosphorylation of GSK3α and GSK3β at S21/S9 was absent in α/β DKI platelets under both basal and stimulated conditions (thrombin, 0.2 U/mL, 5 min). Flow cytometry experiments to determine activation of integrin α_IIb_β_3_ (measured using JON/A-PE, C, E) and α-granule secretion (measured using CD62P-FITC, D, F) revealed responses in α/β DKI platelets were reduced in response to thrombin ((**C**,**D**), *n* = 11) and enhanced in response to CRP-XL ((**E**,**F**), *n* = 8). Median fluorescence values were converted to a % of the maximal matched WT response to thrombin. Curves were fitted with a four-parameter logistic equation (GraphPad Prism 7.04). pEC50 values are indicated in the tables and Student’s *t*-test was used to test for statistical differences (*p*-value indicated in figure). CRP-XL-mediated integrin α_IIb_β_3_ activation max: WT = 74.1 ± 6.5, α/β DKI = 86.9 ± 5.3, *p* = 0.094, *n* = 8. CRP-XL-mediated α-granule secretion max: WT = 36.7 ± 5.6, α/β DKI = 55.3 ± 7.5, *p* = 0.040, *n* = 8. (**C**–**F**). LTA determined that α/β DKI platelets had (**G**) reduced aggregation in response to thrombin (*n* = 4–6) and (**H**) enhanced aggregation in response to CRP-XL (*n* = 3–6). A 2-way ANOVA (Variable 1 = genotype, Variable 2 = concentration of agonist) followed by a Bonferroni’s multiple comparisons test was used to test for differences between groups. * *p*< 0.05.

Immunoblotting for GSK3α and GSK3β with isoform-specific and dual-specificity antibodies confirmed the presence of both GSK3α and GSK3β in both human and mouse platelets (Figure 1A). GSK3α in mouse platelets was found at a higher molecular mass than the human isoform, which is in agreement with murine GSK3α being a larger protein than the human isoform (GSK3A_HUMAN P49840; mass = 50,981 Da and GSK3A_MOUSE Q2NL51; mass = 51,661 Da). Immunoblotting with the dual specificity antibody determined that, while GSK3β was the predominant isoform expressed in both human and mouse platelets (Figure 1A), there was a 2.1 ± 0.5-fold difference (*n* = 4 ± s.e.m) in the expression of the two isoforms in human platelets, whereas there was 10.8 ± 1.4-fold difference (*n* = 4 ± s.e.m) in mouse platelets. These findings correlate with previously reported mRNA and proteomic analyses [25,26,27].

### 2.2. Thrombin-Mediated Responses Are Reduced in Phosphorylation-Resistant GSK3α/β Platelets

To examine the role of GSK3 N-terminal serine phosphorylation in platelets, we used a mouse model in which the codon encoding Ser21 of GSK3α, and Ser9 of GSK3β, was changed to encode non-phosphorylatable alanine residues. These mice had previously been reported to be born at the expected Mendelian frequency and displayed no overt phenotype [28]. Furthermore, the platelet count, platelet volume, and surface expression of major glycoproteins/receptors were unchanged (Table 1). To confirm the absence of GSK3 phosphorylation in platelets, we immunoblotted total platelet lysate with a phospho-specific antibody against GSK3α/β pSer21/9.

Figure 1B demonstrates that the GSK3α/β Ser21/9 to Ala21/9 mutation results in ablation of both basal and agonist-stimulated phosphorylation of GSK3α/β at Ser21/9 without changes in total GSK3α/β expression. In agreement with our previous findings [9], platelets expressing phosphorylation-resistant GSK3α/β have reduced responsiveness to the protease-activated receptor agonist thrombin (Figure 1C,D,G). Activation of integrin α_IIb_β_3,_ which plays a vital role in promoting both platelet–platelet interactions (aggregation), and supporting platelet adhesion to the extracellular matrix, was reduced when stimulated with low/mid-range concentrations of thrombin (Figure 1C). This resulted in a right-shift of the curve but no alteration in the maximal response. Similar changes were observed for thrombin-stimulated exposure of the platelet α-granule marker P-selectin (Figure 1D). In agreement with these findings, platelet aggregation was reduced in response to low but not high concentrations of thrombin (Figure 1G).

### 2.3. GPVI-Mediated Responses Are Enhanced in Phosphorylation-Resistant GSK3α/β Platelets

Alongside thrombin, collagen is another potent activator of platelets ex vivo. However, the role of GSK3 in collagen-mediated platelet function is currently unknown. The reported effects of GSK3 inhibitors on collagen-mediated platelet function range from inhibitory, no effect, to an enhancing effect [8,9,19,20,29]. In this study, we therefore evaluated the role of GSK3α/β phosphorylation downstream of the collagen receptor GPVI, using the selective agonist CRP-XL (cross-linked collagen-related peptide). In contrast to the results with thrombin, platelets expressing phosphorylation-resistant GSK3α/β had significantly enhanced responses to the GPVI agonist collagen-related peptide (CRP-XL). Integrin α_IIb_β_3_ activation was left-shifted (Figure 1E), whereas maximal exposure of P-selectin in response to CRP-XL was markedly enhanced (Figure 1F). In agreement with these findings GPVI-mediated platelet aggregation was also increased (Figure 1H). These results suggest that GSK3α/β phosphorylation is important in restraining platelet function downstream of GPVI.

### 2.4. GSK3 Paralogs Localise to Distinct Locations in Mouse and Human Platelets

**Figure 2:**
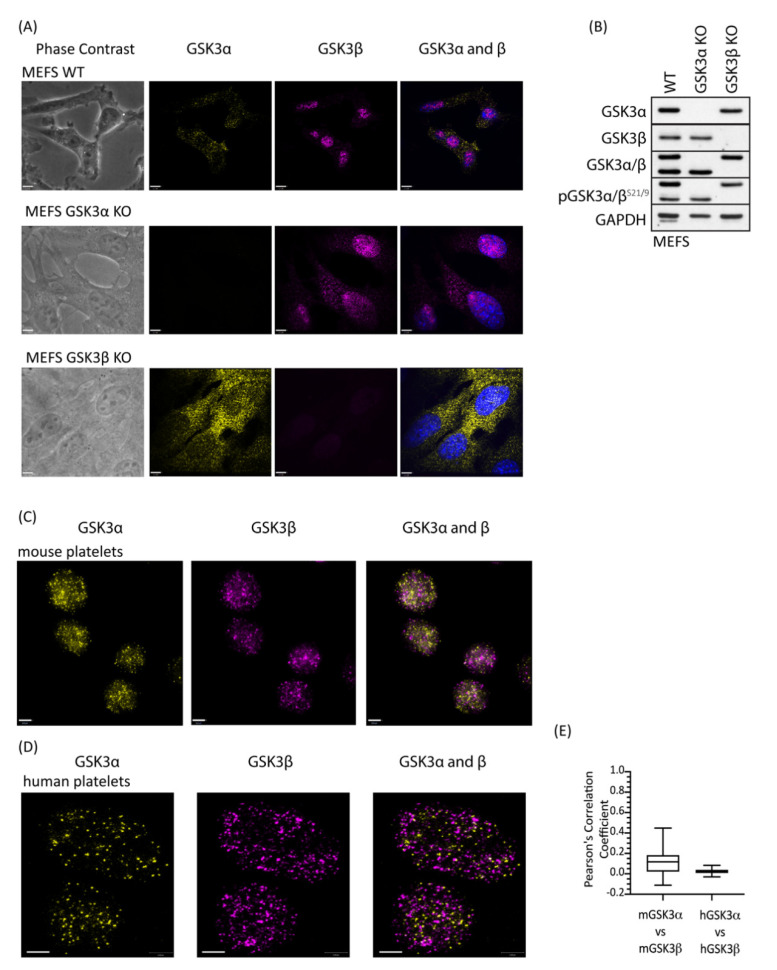
GSK3 paralogs localise to distinct locations in mouse and human platelets. Platelets and MEFs were stained with antibodies specific for GSK3α (D80D1, #4818 CST) and GSK3β (MRU PPU) and imaged using a Leica TCS SP8 STED system: (**A**) Representative images of WT, GSK3α KO and GSK3β KO MEFs stained with antibodies against GSK3α (yellow) and GSK3β (magenta) in the presence of 4′,6-diamidino-2-phenylindole (DAPI, blue) indicated that antibodies used for localisation analyses are specific for GSK3α and GSK3β. Bar represents 6 µm. (**B**) Immunoblotting of WT, GSK3α KO, and GSK3β KO MEFs for GSK3α, GSK3β, and pGSK3α/β^S21/9^ verifies that MEFs have either GSK3α or GSK3β deleted. Data are representative of 3 independent experiments. (**C**) Representative images of mouse platelets showing GSK3α (yellow) and GSK3β (magenta). Bar represents 1 µm. (**D**) Representative images of human platelets showing GSK3α (yellow) and GSK3β (magenta). Bar represents 1 µm. (**E**) Pearson’s correlation coefficient analysis demonstrates that GSK3α and GSK3β are unlikely to interact physically in mouse (mGSK3α vs. mGSK3β, 62 images) and human (hGSK3α vs. hGSK3β, 22 images) platelets.

Although GSK3α and GSK3β are structurally similar they are not functionally identical and have distinct non-redundant roles dictated predominantly by tissue expression [30] but also by subcellular localization [31] and potentially substrate differences [32]. To address whether there is a difference in localisation of the GSK3 paralogs, we performed immunofluorescence on platelets using GSK3α- and GSK3β-specific antibodies. Control studies using GSK3α and GSK3β KO MEFs confirmed that both antibodies were highly specific (Figure 2A, B). Interestingly, GSK3β, but not GSK3α, showed a nuclear localisation in MEFs, which is consistent with previous reports, where active GSK3β accumulates in the nucleus [33,34,35]. Imaging of mouse (Figure 2C) and human (Figure 2D) platelets stained with antibodies specific for GSK3α and GSK3β using super-resolution microscopy (STED) revealed that both isoforms were present in the cytosol in distinct small punctate areas. Interestingly, although the pattern for the two paralogs is very similar, overlay studies confirmed that their localisation is quite distinct. Together, these findings demonstrate distinct and punctate localisation of GSK3α and GSK3β in the cell, which supports the possibility that these GSK3 paralogs may have different substrates and/or functions in platelet activation and thrombosis.

### 2.5. Expression of Phosphorylation-Resistant GSK3α Underlies Enhanced GPVI-Mediated Platelet Activation

**Figure 3:**
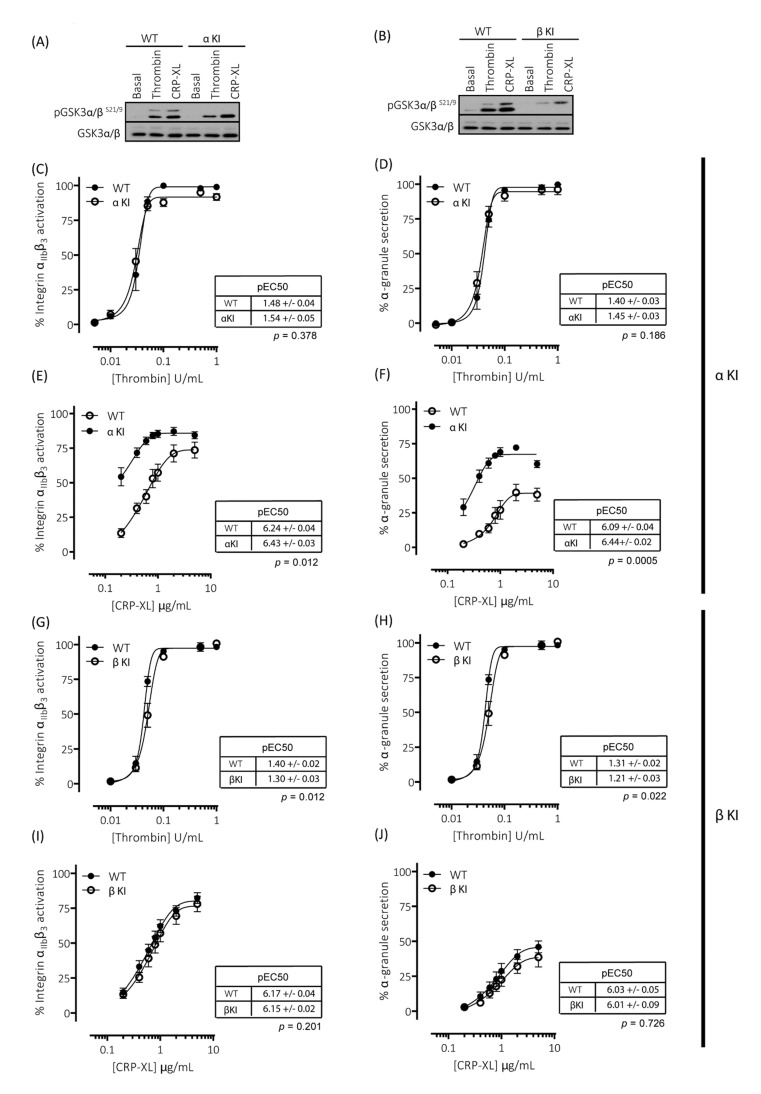
Platelets expressing phosphorylation-resistant GSK3α or GSK3β have altered responses to thrombin and CRP-XL: (**A**) Immunoblotting of platelets from mice expressing phosphorylation-resistant GSK3α (αKI) confirmed the absence of GSK3α phosphorylation at S21 but presence of GSK3β phosphorylation at S9. (**B**) Immunoblotting of platelets from mice expressing phosphorylation-resistant GSK3β (βKI) confirmed the absence of GSK3β phosphorylation at S9 but presence of GSK3α phosphorylation at S21. (**C**–**F**) Flow cytometry experiments to determine activation of integrin α_IIb_β_3_ (measured using JON/A-PE) and α-granule secretion (measured using CD62P-FITC) revealed responses in αKI platelets were unaltered in response to thrombin ((**C**,**D**), *n* = 4) and markedly enhanced in response to CRP-XL ((**E**–**F**), *n* = 7). Flow cytometry experiments revealed responses in βKI platelets were reduced in response to thrombin ((**G**,**H**), *n* = 4) and unaltered in response to CRP-XL ((**I**,**J**), *n* = 7). Median fluorescence values were converted to a % of the maximal matched WT response to thrombin. Curves were fitted with a four-parameter logistic equation (GraphPad Prism 7.04). pEC50 values are indicated in the tables and Student’s *t*-test was used to test for statistical differences (*p*-value indicated in figure). The CRP-mediated maximal responses are as follows: (**E**) α_IIb_β_3_ activation: WT = 76.8 ± 6.8, αKI = 86.9 ± 5.3, *p* = 0.14, *n* = 4. (**F**) α-granule secretion: WT = 40.3 ± 5.3, αKI = 67.4 ± 1.9, *p* = 0.02, *n* = 4. (**I**) α_IIb_β_3_ activation: WT = 84.6 ± 4.4, βKI = 77.6 ± 4.7, *p* = 0.07, *n* = 7. (**J**) α-granule secretion: WT = 47.2 ± 4.6, βKI = 35.3 ± 4.3, *p* = 0.03, *n* = 7.

To evaluate the individual contribution of Ser21 phosphorylation of GSK3α and Ser9 phosphorylation of GSK3β in regulating platelet function, we generated homozygous GSK3 single knock-in mice. These mice, with either Ser21 of GSK3α or Ser9 of GSK3β mutated to Ala, are termed GSK3α knock-in (αKI) and GSK3β knock-in (βKI), respectively. In the GSK3α knock-in platelets, both basal and agonist-stimulated phosphorylation of GSK3α at Ser21 was absent but GSK3β phosphorylation at Ser9 was present without changes in total GSK3α/β expression (Figure 3A). The converse results were observed in GSK3β knock-in platelets (Figure 3B). Measurements of both integrin α_IIb_β_3_ activation and P-selectin exposure revealed that expression of phosphorylation-resistant GSK3α did not affect thrombin-induced platelet activation (Figure 3C,D), but significantly enhanced GPVI-mediated integrin α_IIb_β_3_ activation and P-selectin exposure (Figure 3E,F). In agreement with the findings in GSK3 double knock-in (α/β DKI) platelets, maximal integrin α_IIb_β_3_ activation was not significantly increased, but the concentration response curve was shifted to the left and maximal P-selectin exposure was markedly increased. These results suggest that the enhancement in CRP-XL-mediated responses observed in phosphorylation-resistant GSK3α/β (α/β DKI) platelets was due to the absence of phosphorylation at Ser21 on GSK3α. Together, these findings indicate that phosphorylation of GSK3α at Ser21 is a requirement for restraining GPVI-mediated platelet activation.

### 2.6. Expression of Phosphorylation-Resistant GSK3β Underlies Reduced Thrombin-Mediated Platelet Activation

Measurements of both integrin α_IIb_β_3_ activation and P-selectin exposure revealed that expression of phosphorylation-resistant GSK3β resulted in reductions in both integrin α_IIb_β_3_ activation and P-selectin exposure induced by low/mid-range concentrations of thrombin (Figure 3G,H). These findings are in agreement with those previously published whereby platelets lacking an allele of GSK3β are hypersensitive to PAR-mediated platelet activation [19]. In contrast, GPVI-mediated integrin α_IIb_β_3_ activation and P-selectin exposure was unaltered in GSK3β KI platelets (Figure 3I,J). These findings therefore strongly indicate that phosphorylation of GSK3β at Ser9 supports PAR-mediated platelet activation.

### 2.7. Intracellular Ca^2+^ Signalling Is Unaltered in Platelets Expressing Phosphorylation-Resistant GSKα or GSKβ

**Figure 4:**
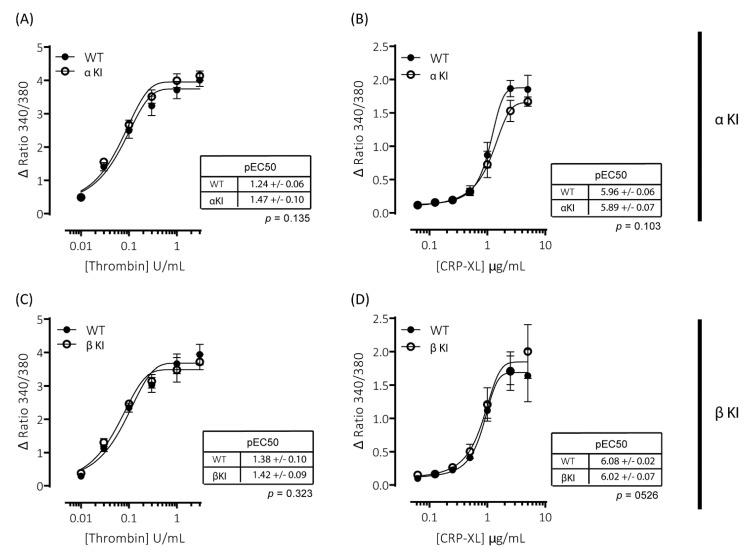
Intracellular Ca^2+^ signalling is unaltered in platelets expressing phosphorylation-resistant GSK3α or GSK3β. Changes in intracellular [Ca^2+^] were determined by loading platelets with the calcium indicator Fura-PE3 AM and monitoring fluorescence at 340/380 nm on an Infinite^®^ 200 PRO multimode reader (TECAN, Reading, UK). Platelets from αKI mice had no significant alterations in calcium mobilisation in response to (**A**) thrombin (*n* = 4) or (**B**) CRP-XL (*n* = 6). Platelets from βKI mice had no significant alterations in calcium mobilisation in response to (**C**) thrombin (*n* = 3) or (**D**) CRP-XL (*n* = 4). Data are expressed as the change (minus baseline) in ratio 340/380 nm. Curves were fitted with a four-parameter logistic equation (GraphPad Prism 7.04). pEC50 values are indicated in the tables and Student’s *t*-test was used to test for statistical differences (*p*-value indicated in figure).

Agonist-induced increases in cytosolic Ca^2^^+^ are essential for platelet activation and regulate both integrin α_IIb_β_3_ activation and granule secretion. Due to the alterations in platelet function observed in platelets expressing either phosphorylation-resistant GSK3α (αKI) or GSK3β (βKI), we examined whether thrombin and/or CRP-XL-mediated increases in intracellular [Ca^2+^] were altered using the intracellular Ca^2+^ indicator FURA-PE3. Stimulation of mouse platelets with thrombin or CRP-XL elicited concentration-dependent increases in intracellular [Ca^2+^]; however, we observed no significant alterations in the responses in either platelets expressing phosphorylation-resistant GSK3α (αKI, Figure 4A,B) or GSK3β (βKI, Figure 4C,D).

### 2.8. Pathways Involved in Amplification of Platelet Function Are Unaltered in Platelets Expressing Phosphorylation-Resistant GSKα or GSK3β

**Figure 5:**
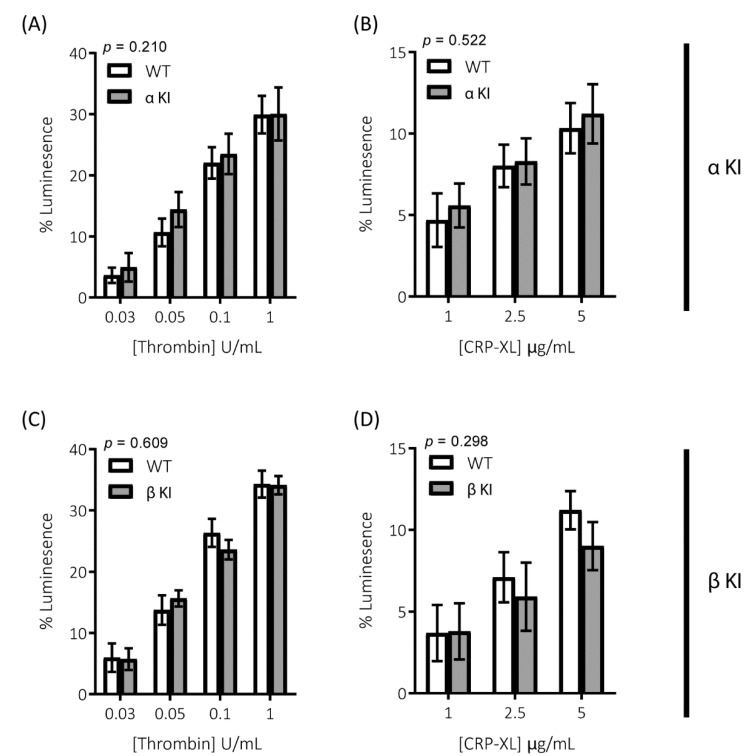
Secretion of the δ-granule cargo ATP is unaltered in platelets expressing phosphorylation-resistant GSK3α or GSK3β. ATP secretion from platelets was monitored using CHRONO-LUME^®^ and the Infinite^®^ 200 PRO multimode reader (TECAN, Reading, UK). Platelets from αKI mice had no significant alterations in ATP secretion in response to (**A**) thrombin or (**B**) CRP-XL (*n* = 5). Platelets from βKI mice had no significant alterations in ATP secretion in response to (**C**) thrombin or (**D**) CRP-XL (*n* = 5). A 2-way ANOVA (Variable 1 = genotype, Variable 2 = concentration of agonist) was used to test for differences between groups. Data are the peak luminescence expressed as a % of luminescence signal evoked by a fixed concentration of ATP.

**Figure 6:**
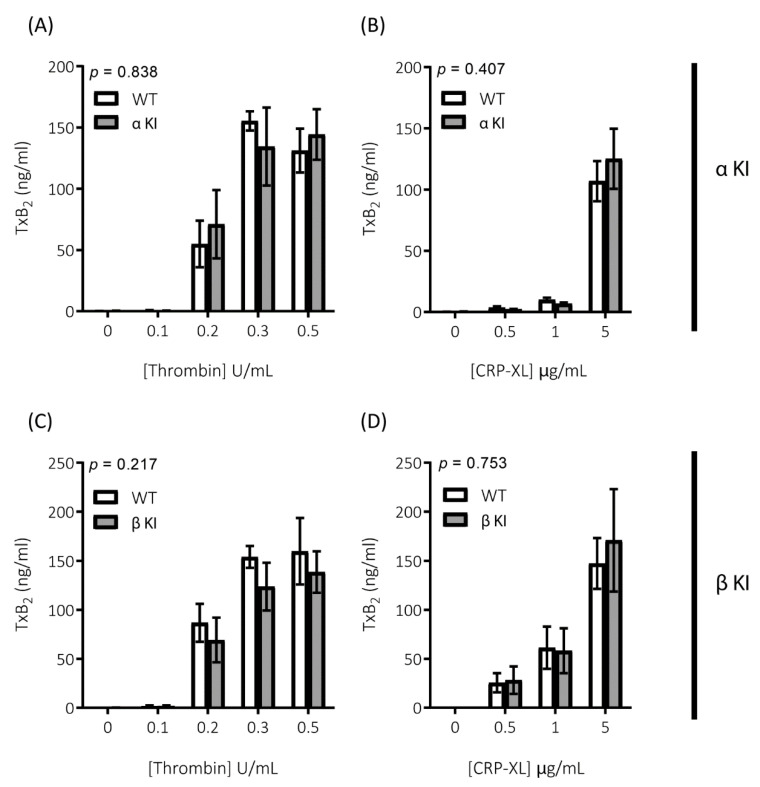
Generation of TxA_2_ is unaltered in platelets expressing phosphorylation-resistant GSK3α or GSK3β. TxA_2_ generation was determined by measuring the concentration of TxB_2_ in platelet supernatants using a TxB_2_ ELISA kit. Platelets from αKI mice had no significant alterations in TxA_2_ generation in response to (**A**) thrombin or (**B**) CRP-XL (n = 4). Platelets from βKI mice had no significant alterations in TxA_2_ generation in response to (**C**) thrombin or (**D**) CRP-XL (n = 4). A 2-way ANOVA (Variable 1 = genotype, Variable 2 = concentration of agonist) was used to test for differences between groups. *p*-values are indicated in figure. Data are expressed as ng/mL TxB_2_ determined from standard curve.

**Figure 7:**
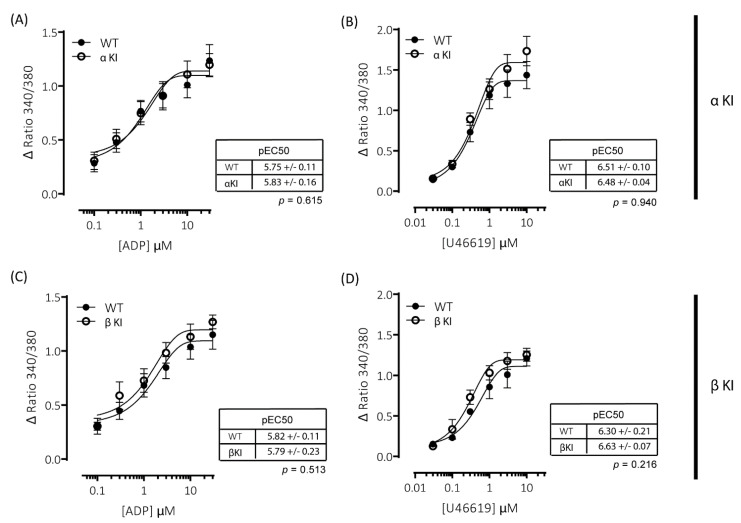
ADP- and U46619-elicited changes in intracellular [Ca^2+^] are comparable between wild-type and phosphorylation-resistant GSK3α or GSK3β platelets. Changes in intracellular [Ca^2+]^ were determined by loading platelets with the calcium indicator Fura-PE3 AM and monitoring fluorescence at 340/380 nm on an Infinite^®^ 200 PRO multimode reader (TECAN, Reading, UK). Platelets from αKI mice had no significant alterations in calcium mobilisation in response to (**A**) ADP (pEC50: *p* = 0.74, *n* = 6) or (**B**) U46619 (pEC50: *p* = 0.43, *n* = 6). Platelets from βKI mice had no significant alterations in calcium mobilisation in response to (**C**) ADP (pEC50: *p* = 0.86, *n* = 3) or (**D**) U46619 (pEC50: *p* = 0.10, *n* = 3). Data are expressed as the change (minus baseline) in ratio 340/380 nm. Curves were fitted with a four-parameter logistic equation (GraphPad Prism 7.04). pEC50 values are indicated in the tables and Student’s *t*-test was used to test for statistical differences (*p*-values indicated in figure).

Platelets rely on endogenous secondary signal amplification mechanisms to achieve a relevant level of response to vascular injury. Two major amplification pathways are the secretion of δ-granules containing ADP/ATP and the activation of cyclooxygenase and subsequent generation of thromboxane (TxA_2_). The alterations in platelets expressing either phosphorylation-resistant GSK3α or GSK3β could be due to modifications in either the release/generation of these mediators or the response to them. Monitoring the release of ATP with CHRONO-LUME^®^, however, revealed no significant alterations in δ-granule secretion in either phosphorylation-resistant GSK3α (αKI) or GSK3β (βKI) platelets (Figure 5A–D). We also observed no significant alterations in TxA_2_ generation (Figure 6A–D). Furthermore, increases in cytosolic Ca^2+^ evoked by ADP or the TxA_2_ analogue U46619 were also unaltered in phosphorylation-resistant GSK3α and GSK3β platelets (Figure 7A–D). These data determine that expression of phosphorylation-resistant GSK3α or GSK3β does not alter the release/generation of major secondary mediators or the response to them.

### 2.9. Role of GSK3α/β Phosphorylation in Thrombus Formation

**Figure 8:**
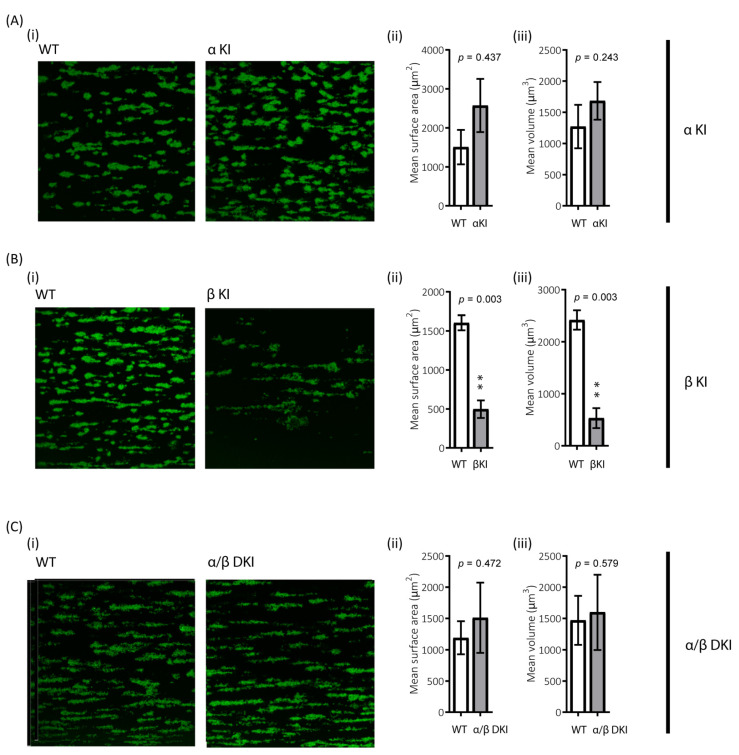
Reduced platelet accumulation on collagen under shear in blood from phosphorylation-resistant GSK3β mice. Whole anticoagulated blood was flowed at 1000 s^−1^ over fibrillar collagen coated Ibidi^®^ μ-slides (for parallel flow assays): (**A**) Blood from αKI mice exhibited similar amount of platelet deposition compared to WT controls (*n* =12). In contrast (**B**) Blood from βKI mice exhibited reduced platelet deposition compared to WT controls (*n* = 7). (**C**) Blood from α/β DKI mice exhibited similar amount of platelet deposition compared to WT controls (*n* = 10). Thrombus formation was quantified from fixed samples (end-point) by imaging 5 randomly chosen fields of view using a Leica SP5-II confocal laser scanning microscope attached to a Leica DMI 6000 inverted epifluorescence microscope (Leica Microsystems, United Kingdom). Data are representative images, and quantification of surface area covered μm^2^ and total thrombus volume μm^3^. Student’s *t*-test was used to test for statistical differences between genotypes **: *p* < 0.01.

The role of platelets in haemostasis/thrombosis critically relies on their ability to form stable aggregates, i.e., thrombi, under shear. To examine the role of GSK3α/β phosphorylation in this process, the ability of platelets to adhere to collagen under arterial shear was evaluated. There was a trend for enhanced thrombus volume and surface coverage after flowing blood from phosphorylation-resistant GSK3α mice (αKI) for 2 min over collagen, although this did not reach significance (Figure 8A). However, platelet accumulation on collagen in blood from phosphorylation-resistant GSK3β (βKI) was largely impaired (Figure 8B). Interestingly, platelet accumulation on collagen using blood from GSK3 double knock-ins (ki/ki) was unaltered, demonstrating that the absence of GSK3α phosphorylation can compensate for the reduction in thrombus formation observed in the GSK3β knock-ins (Figure 8C).

## 3. Discussion

In this study, we investigated the role of agonist-mediated inhibitory GSK3 phosphorylation in regulating platelet function and thrombosis using a phosphorylation-resistant GSK3 knock-in mouse model. Surprisingly, we found divergent roles for phosphorylation of GSK3α at Ser21 and GSK3β at Ser9. The mutation of GSK3α at Ser21 to Ala enhanced platelet activation, whereas the mutation of GSK3β at Ser9 to Ala supressed platelet activation.

The stimulation of platelets with various agonists results in the activation of signalling pathways, leading to the phosphorylation of GSK3α/β at Ser21/9 and reducing its activity against “primed” (pre-phosphorylated) substrates. Logic suggests that, if stimuli that promote platelet function result in the phosphorylation/inhibition of GSK3, GSK3 is a negative regulator of platelet function. This idea has been supported by previous findings using GSK3 inhibitors and a single-allele knock-out mouse. Various GSK3 inhibitors have been shown to hypersensitise platelets, enhancing aggregation in response to PAR and CLEC-2 activation [9,36] and rescuing defective thrombus formation in PI3Kβ KO mice [22]. Furthermore, GSK3β^+/-^ platelets were found to be hypersensitive to PAR4-AP and the GSK3β^+/-^ mice had increased sensitivity to thrombotic insult [19]. Our results with the phosphorylation-resistant GSK3 mice support these findings insofar as when GSK3 cannot be inhibited through n-terminal serine phosphorylation, platelet function in response to PAR activation is suppressed. Furthermore, we identify that it is specifically the phosphorylation of GSK3β, and not GSK3α, that is required to support PAR-mediated platelet function.

In direct contrast, we observed that platelets from phosphorylation-resistant GSK3α mice were hypersensitive to stimulation with the GPVI agonist CRP-XL. This was surprising, as we had previously found the GSK3 inhibitor CHIR99021 to have little effect on CRP-XL-induced platelet aggregation whilst enhancing PAR-mediated platelet aggregation [9]. However, other studies have reported effects of GSK3 inhibitors on GPVI-mediated events. Pre-incubation of platelets with lithium (competitive inhibitor of Mg^2+^ and increasing GSK3 phosphorylation), TDZD-8 (non-ATP competitive), or SB415286 (ATP competitive) inhibited collagen-mediated platelet aggregation [20]. SB216763 (ATP competitive) was also shown to inhibit collagen-induced platelet activation but enhanced platelet aggregation induced by a subthreshold concentration of thrombin [8]. These results would fit with our findings and suggests that GSK3 can play differential roles in different platelet activation pathways. However, in the course of our study, we established that it was not necessarily that GSK3 plays differential roles but that the different paralogs are mainly involved in regulating different platelet activation pathways, as we identified that it is the phosphorylation of GSK3α, and not GSK3β, that is required to suppress GPVI-mediated platelet function. One mechanism by which GSK3α phosphorylation may limit CRP-mediated integrin activation is by enhancing procoagulant activity; the latter is known to be associated with integrin inactivation [37,38]. Although we cannot rule out that phosphorylation of GSK3α affects procoagulant activity, the latter requires much stronger platelet stimulation, with high concentrations of GPVI agonists and thrombin required, suggesting that an alteration in this balance would have a limited effect on CRP-mediated integrin activation under our conditions.

In our study, we also performed in vitro flow experiments under high shear and found that thrombus formation was severely impaired in the GSK3β KI mice. This is an intriguing finding as CRP-mediated Ca^2+^ signalling, integrin activation, and granule secretion were unaffected, suggesting that initial adhesion to collagen under flow is impaired in GSK3β KI platelets. GPVI dimerization is important for collagen-mediated adhesion and platelet function, especially under flow, and one potential mechanism may therefore be that GPVI dimerization is regulated by GSK3β. This would agree with the findings in human platelets that GPVI dimerization is an active process that is enhanced by positive feedback pathways and can be inhibited by cAMP-mediated signalling pathways [39]. The lack of anti-mouse GPVI dimer specific antibodies prevented us from studying this further. In addition to GPVI, interaction of GPIb with vWF also contributes to platelet adhesion and thrombus formation under flow [40]. We did, however, not observe alterations in GPIb receptor expression level, although this does not rule out changes in GPIb-mediated interactions and/or signalling. Future work will have to elucidate whether impaired GPIb function may contribute to the βKI phenotype. Interestingly, the impaired thrombus formation under flow was rescued by phosphorylation resistance of GSK3α, revealing that, under these conditions, GSK3a and GSK3β can compensate each other. However, whether this is through the same mechanism is presently unclear.

Although GSK3α and GSK3β are paralogs and therefore structurally similar, they are not functionally identical. This was first highlighted when it was found that the GSK3β knock-out mouse is embryonic lethal, whereas the GSK3α knock-out is viable. There is now a growing body of evidence that they have distinct non-redundant roles that are dictated by tissue expression, subcellular localisation, and potential substrate differences [41,42,43,44,45,46]. Both human and mouse platelets express both paralogs; therefore, paralog expression is unlikely to explain the diverging effects observed in phosphorylation-resistant GSK3 platelets. In contrast, we report that GSK3α and GSK3β are localized to distinct sites within human and mouse platelets and therefore unlikely to interact physically. It is possible that this localisation modulates GSK3α and GSK3β differentially through the local signalling environment and by restricting the potential substrate pool. Regarding GSK3 substrates, there is growing recognition that certain substrates are preferentially linked to one isoform. However, whether this is due to differences in kinase–substrate binding affinity between the isoforms is yet to be established. It is more likely that multiple regulatory mechanisms dictate substrate discretion such as substrate availability (pre-phosphorylation), localisation, and association with other proteins.

How the alterations in GSK3 phosphorylation lead to changes in platelet activation is presently unclear. We determined that they occurred without significant changes in mobilisation of intracellular Ca^2+^ or in pathways leading to the production/release and response to the major secondary mediators. Future work examining GSK3 interactors and substrates will likely yield further insight into this. This will need to take into consideration some important caveats regarding serine phosphorylation of the GSK3α/β in that the phosphorylation is a competitive inhibition and that increasing the concentration of substrate can therefore out-compete the phosphorylated pseudo-substrate domain. Furthermore, GSK3 substrates require prior phosphorylation (priming) to permit phosphorylation by GSK3. Therefore, if platelet stimulation resulted in inhibition of the priming kinase then substrate regulation may persist in the phosphorylation-resistant mice. Additionally, as phosphorylation is a reversible process, with regulation of phosphatases playing a key role in signalling pathways, it is possible that platelet activation of a phosphatase is sufficient to decrease GSK3 substrate phosphorylation. This makes it challenging to predict the outcome of GSK3 phosphorylation on the phosphorylation status of their substrates.

This study demonstrates opposing roles for GSK3α and GSK3β in platelet function and highlights the complexity of signalling downstream of PI3kinase/Akt/GSK3 in regulating platelet function. Identification of GSK3 interactors and substrates will guide future work to elucidate their underlying mechanism.

## 4. Materials and Methods

### 4.1. Materials

Akt (#2920), phospho-Akt^T308^ (#13038), phospho-Akt^S473^ (#4060), GSK3α (#4337), GSK3α (#4818), GSK3β (#12456), GSK3α/β (#5676), phospho-GSK3α/β^S21/9^ (#8566), GS (#3839), phospho-GS^S641^ (#3891), LAT (#9166), phospho-LAT^Y191^ (#3584), PLCγ2 (#3872), phospho-PLCγ2^Y759^ (#3874), Syk (#2717) and phospho-Syk^Y525/526^ (#2711) antibodies were from Cell Signalling Technologies (New England Biolabs, Hitchin, UK). RAP1 (SC-65) and GAPDH (SC-25778) antibodies were from Santa Cruz Biotechnology (Insight Scientific, Middlesex, UK). GSK3β antibody was from MRC PPU Reagents and Services (Dundee, UK). FITC-conjugated glycoprotein VI (GPVI, JAQ1), FITC-conjugated CD49b (Sam.C1), FITC-conjugated CD42b (Xia.G5), PE-conjugated integrin α_IIb_β_3_ (JON/A) and FITC-conjugated CD62P (WugE9) anti-mouse antibodies were from Emfret ANALYTICS (Wurzberg, Germany). FITC-conjugated CD41 (MWReg30) anti-mouse antibody was from AbD Serotec (Kidlington, UK). Chrono-Lume^®^ was from Chrono-log Corporation (Labmedics, Abingdon, UK). Cross-linked collagen-related peptide (CRP-XL) was synthesized by Prof. Richard Farndale (Department of Biochemistry, University of Cambridge, UK). Collagen Reagens HORM^®^ Suspension was from Takeda (Linz, Austria). Enhanced chemiluminescent detection reagents were from GE Healthcare (Amersham, UK). Peroxidase-conjugated and far-red/infrared secondary antibodies were from Jackson Immunoresearch (Stratech Scientific Ltd., Ely, UK). NuPAGE SDS–PAGE sample buffer was from Invitrogen (Paisley, UK). cOmplete™ protease inhibitor cocktail tablets were from Roche (West Sussex, UK). All other reagents were from Sigma (Poole, UK), unless otherwise indicated.

### 4.2. Mice

Animal studies were approved by local research ethics and mice bred for this purpose under a U.K. Home Office project license (PPL30/3445). The phosphorylation-resistant GSK3α/β “knock-in” line was generated as previously described [28] and backcrossed to C57BL/6 for 9 generations before cryopreservation. To investigate the role of GSK3α/β phosphorylation in regulating platelet function GSK3α^S21A/S21A^ (αKI), GSK3β^S9A/S9A^(βKI), GSK3α/β^S21A/S21A/S9A/S9A^ (α/β DKI), and control wild-type GSK3α/β^+/+/+/+^ (WT) mice were bred and genotyped as previously described [28]. Experiments were performed on age- and sexed-matched mice.

### 4.3. Platelet Isolation

Mice (8–16 weeks old) were sacrificed by rising CO_2_ inhalation, in accordance with Schedule 1 of the Animals (Scientific Procedures) Act (1986), and blood was drawn by cardiac puncture into a syringe containing 4% trisodium citrate (1:9, *v*/*v*). Human blood was obtained with approval from the local Research Ethics Committee of the University of Bristol from healthy drug-free volunteers, who gave full informed consent in accordance with the Declaration of Helsinki. Washed platelets were obtained as previously described [47] and resuspended at 4 × 10^8^/mL in modified HEPES-Tyrode buffer (145 mM NaCl, 3 mM KCl, 0.5 mM Na_2_HPO_4_, 1 mM MgSO_4_, 10 mM HEPES, pH 7.2, 0.1% (*w*/*v*) D-glucose, 0.02 U/mL apyrase, and 10 μM indomethacin).

### 4.4. Protein Extraction and Immunoblotting

Platelets (4 × 10^8^/mL) were incubated with agonists as indicated. Platelet suspensions were lysed directly in 4× NuPAGE sample buffer containing 0.5 M DTT. Lysates were analysed by SDS-PAGE/Western blotting using bis-tris gels as previously described [47] and visualised by either enhanced chemiluminescence or near-infrared detection (Odyssey^®^CLx, LI-COR Biotechnology UK Ltd., Cambridge, UK)). For quantification of immunoblotting, densitometry was performed using ImageJ software (NIH) or LI-COR Image Studio^TM^ Software (Biotechnology UK Ltd., Cambridge, UK).

### 4.5. Flow Cytometry

Flow cytometry analyses were performed as previously described [21]. Integrin αIIbβ3 activation and α-granule secretion were measured using a PE-conjugated antibody (JON/A) directed against the high affinity form of integrin αIIbβ3 and a FITC-conjugated antibody (Wug.E9) for the α-granule marker CD62P (P-selectin), respectively. Surface expression of integrin α_IIb_ (CD41), integrin α2 (CD49b), GP1bα (CD42b), and GPVI were measured in resting platelets with FITC-conjugated antibodies against CD41 (MWReg30), CD49b (Sam.C1), CD42b (Xia.G5), and GPVI (JAQ1), respectively. Samples were fixed with 1 % paraformaldehyde for 30 min and 10,000 platelet events per sample recorded on a BD^TM^ LSR II or BD Accuri^TM^ C6 plus (BD Biosciences, Oxford, UK).

### 4.6. Platelet Aggregation

Agonist-stimulated aggregation of washed platelets (2 × 10^8^/mL) was monitored in a Chronolog 590-2A aggregometer (Labmedics, Abingdon, UK) at 37 °C under stirring conditions as previously described [21].

### 4.7. Localisation of GSK3 Isoforms

Fixed platelets or mouse embryonic fibroblasts (MEFs) were permeabilized and stained with GSK3α (D80D1) XP^®^ rabbit mAb (#4818, Cell Signalling Technologies) and/or GSK3β sheep pAb (MRC PPU, Dundee, UK), followed by staining with secondary antibodies conjugated to Alexa Fluor-488 and/or Alexa Fluor-568 (ThermoFisher Scientific, Loughborough, UK). STED imaging of samples was achieved as previously described using a Leica TCS SP8 STED system [48].

### 4.8. Intracellular Ca^2+^ Measurements

Changes in cytosolic [Ca^2+^]i were measured in washed platelets (2 × 10^8^/mL) loaded with Fura-PE3 in the presence of 1 mM CaCl_2_. Ratiometric measurements (340/380 nm) were taken using an Infinite^®^ 200 PRO multimode reader (TECAN, Reading, UK).

### 4.9. δ-Granule Secretion

Agonist-stimulated δ-granule secretion was assessed by monitoring ATP release from platelets (2 × 10^8^/mL) using a luciferin/luciferase reagent (CHRONO-LUME^®^, Labmedics, Abingdon, UK). Luminescence was measured using an Infinite^®^ 200 PRO multimode reader (TECAN, Reading, UK).

### 4.10. TxA_2_ Generation

TxA_2_ generation was assessed by measuring TxB_2_ in platelet supernatants using a commercially available colorimetric ELISA kit (Enzo Life Sciences, Exeter, UK) as previously described [49].

### 4.11. In Vitro Thrombus Formation

Whole blood thrombus formation was performed as previously described [50] using Ibidi^®^ μ-Slide VI0.1 flow chambers (Thistle Scientific Ltd., Glasgow, UK) connected to an Aladdin AL-1000 syringe pump (World Precision Instruments, UK). Anticoagulated (40 μM PPACK and 2 U/mL heparin) fluorescently labelled (2 μM DiOC_6_, 10 min) blood was perfused at a constant shear rate of 1000 s^− 1^ for 2 min over collagen (50 μg/mL fibrillar HORM^®^ collagen). Samples were fixed, and images were captured on a Leica SP5-II CLSM attached to a Leica DMI 6000 inverted epifluorescence microscope. Quantification of surface coverage and thrombus volume was performed using Volocity^®^ 6.1.1 (Perkin Elmer Inc., San Jose, CA, USA).

### 4.12. Data Analysis

Data were analysed and fitted using GraphPad Prism 7.04 (San Diego, CA, USA). All data are presented as the mean ± s.e.m of at least three independent observations. Data presented with statistical analysis were tested as described in the figure legends.

## Figures and Tables

**Table 1:** Whole blood counts and platelet surface receptor expression in wild-type and phosphorylation-resistant GSK3α/β mice. Complete blood counts were conducted using a Pentra ES60 (Horiba) and adjusted for anticoagulant volume. Surface expression of integrin α_IIb_ (CD41), integrin α2 (CD49b), GP1bα (CD42b), and GPVI were measured in resting platelets by flow cytometry using FITC-conjugated antibodies. Wilcoxon tests were used to test for statistical differences between genotypes.

	WT		α/β DKI			
	Mean	s.e.m	Mean	s.e.m	*n*	*p*-Value
Platelets 10^3^/mm^3^	903	107	744	52	9	0.48
MPV mm^3^	5.31	0.05	5.48	0.14	9	0.38
WBC 10^3^/mm^3^	10.51	1.10	8.87	1.04	9	0.42
RBC 10^6^/mm^3^	10.06	0.24	9.37	0.30	9	0.06
Integrin α_II_ mfi	9672	882	8793	1000	7	0.46
Integrin α_2_ mfi	896	87	884	79	7	>0.99
GP1bα mfi	4498	439	4346	705	7	0.84
GPVI mfi	1211	89	1397	135	7	0.38
